# Epigenetic suppression of creatine kinase B in adipocytes links endoplasmic reticulum stress to obesity-associated inflammation

**DOI:** 10.1016/j.molmet.2024.102082

**Published:** 2024-12-13

**Authors:** Gianluca Renzi, Ivan Vlassakev, Mattias Hansen, Romane Higos, Simon Lecoutre, Merve Elmastas, Ondrej Hodek, Thomas Moritz, Lynn M. Alaeddine, Scott Frendo–Cumbo, Ingrid Dahlman, Alastair Kerr, Salwan Maqdasy, Niklas Mejhert, Mikael Rydén

**Affiliations:** 1 Department of Medicine (H7), Karolinska Institutet, ME Endokrinologi, Karolinska University Hospital Huddinge, SE-141 83, Huddinge, Sweden; 2 Nutrition and Obesities: Systemic Approaches Research Group (Nutri-Omics), Sorbonne Université, INSERM, F-75013 Paris, France; 3 Swedish Metabolomics Center, Department of Forest Genetics and Plant Physiology, Swedish University of Agricultural Sciences, Umeå, Sweden; 4 The Novo Nordisk Foundation Center for Basic Metabolic Research, Faculty of Health and Medical Sciences, University of Copenhagen, Copenhagen, Denmark; 5 Department of Clinical Science and Education, Karolinska Institutet, Stockholm, Sweden; 6 Steno Diabetes Center, Copenhagen, Herlev, Denmark

**Keywords:** Creatine pathway, Glycolysis, Immunometabolism, Tunicamycin, Chromatin remodeling

## Abstract

In white adipose tissue, disturbed creatine metabolism through reduced creatine kinase B (CKB) transcription contributes to obesity-related inflammation. However, the mechanisms regulating *CKB* expression in human white adipocytes remain unclear. By screening conditions perturbed in obesity, we identified endoplasmic reticulum (ER) stress as a key suppressor of *CKB* transcription across multiple cell types. Through follow-up studies, we found that ER stress through the IRE1–XBP1s pathway, promotes *CKB* promoter methylation via the methyltransferase DNMT3A. This epigenetic change represses *CKB* transcription, shifting metabolism towards glycolysis and increasing the production of the pro-inflammatory chemokine CCL2. We validated our findings in vivo, demonstrating that individuals living with obesity show an inverse relationship between *CKB* expression and promoter methylation in white adipocytes, along with elevated CCL2 secretion. Overall, our study uncovers a regulatory axis where ER stress drives inflammation in obesity by reducing CKB abundance, and consequently altering the bioenergetic state of the cell.

## Highlights

• ER stress represses *CKB* transcription and induces inflammation in adipocytes.
• IRE1-XBP1s downregulates *CKB* via epigenetic mechanisms.
• XBP1s interacts with DNMT3A to induce DNA methylation at the *CKB* promoter.
• Restoring *CKB* expression normalizes ER stress-driven inflammation.
• In obesity, *CKB* promoter methylation associates with *CKB* gene expression and adipose inflammation.

## 1 Introduction

Obesity, a metabolic disorder linked to insulin resistance and type 2 diabetes, is characterized by an accumulation of white adipose tissue (WAT) marked by a chronic low-grade inflammation [[1], [2], [3], [4]]. This milieu is partly initiated by metabolic rewiring in adipocytes, which triggers the production of cytokines and chemokines, including C–C motif chemokine ligand 2 (CCL2), promoting immune cell infiltration [2,5]. By studying this immunometabolic crosstalk, we recently showed that disturbed creatine metabolism in human and murine white fat cells is an important mediator in this process [2]. More specifically, our data demonstrated that reduced expression of Creatine Kinase B (CKB) in the obese state led to a metabolic switch favoring glucose utilization which in turn promoted CCL2 secretion. However, the mechanisms regulating white adipocyte *CKB* expression in the overweight/obese state remain unclear.

Herein, we screened for potential regulators of *CKB* expression in white adipocytes. We focused on processes known to be perturbed in WAT from individuals living with obesity and insulin resistance. This revealed that activation of endoplasmic reticulum (ER) stress results in a pronounced reduction of CKB mRNA and protein via XBP1s-DNMT3A-mediated methylation of the proximal *CKB* promoter. Through orthogonal approaches, we show that perturbations of this axis alter CCL2 production. Altogether, our results reveal a link between ER stress, creatine metabolism and pro-inflammatory pathways in white adipocytes.

## 2 Materials and methods

### 2.1 Materials

Materials, e.g., compounds, antibodies and primers, used in this study are listed in Table S1.

### 2.2 Human cohorts

Subcutaneous adipose tissue biopsies were obtained from the periumbilical area by needle aspiration under local anesthesia. Cohort 1 included 39 women living with (n = 19) or without (n = 20) obesity and is part of a larger study registered at clinicaltrials.gov (NCT01727245). Relevant clinical parameters can be found in Table S2. In the present work, it was used to determine mRNA expression by qPCR. Cohort 2 has been described previously and consists of women living with (n = 8) or without obesity (n = 8) where subcutaneous adipose tissue gene expression and adipocyte DNA methylation profiles are available [6].

### 2.3 Cell culture

#### 2.3.1 Human adipocyte progenitors cells

Human adipocytes were differentiated from CD55^+^ progenitor cells isolated from abdominal subcutaneous WAT of one male donor [7]. In brief, cells were cultured using DMEM media containing 10% FBS, HEPES (10 mmol/l), Penicillin-Streptomycin (50 mg/ml) supplemented with Fibroblast growth factor-2 (FGF2, [2.5 ng/ml]). To induce differentiation, cells were plated at 90% confluency. FGF was removed after 24 h and differentiation induced as previously described [2].

#### 2.3.2 3T3-L1 and HEK293 cells

HEK293 cells were cultured using GlutaMAX DMEM media supplemented with 10% heat inactivated FBS and HEPES (10 mmol/l). Cells were seeded at 90% confluency in six-well plates, (approximately 1 × 10^6^ cells/well) and used for lentiviral generation as described below.

3T3-L1 cells were cultured in 10% bovine calf serum in growth media consisting of DMEM, l-glutamine (2 mmol/l), Penicillin-Streptomycin (100 mg/ml). Once cells reached confluence, a standard protocol was used to induce differentiation. In brief, cells were incubated in a medium containing a dexamethasone (1 μmol/l), IBMX (100 μmol/l), insulin (1.75 nmol/l) and rosiglitazone (BRL) (10 μmol/l) for three days. Following this, the media was replaced with growth media plus 10% FBS and insulin for two more days. All experiments were carried out seven days after induction of differentiation.

#### 2.3.3 Screening for regulators of CKB expression

To screen for CKB regulators, human adipocytes were treated with various compounds after 13 days of differentiation. The treatments included dimethyloxalylglycine (200 μmol/l) for 24 h, TNFα (1 ng/ml) for 24 h, epinephrine (1 μmol/l) for 3 h, menadione (30 μmol/l) for 3 h, tunicamycin (5 ng/ml) for 8 h and palmitate (1 mmol/l) for 24 h. To make the cells insulin-responsive, they were incubated with differentiation media containing 1/20 of the normal insulin concentration for 48 h, followed by insulin starvation for an additional 24 h. Cells were then stimulated with insulin (50 nmol/l) for 2 h. The concentrations used were based on published studies [[8], [9], [10], [11], [12], [13]].

#### 2.3.4 RNAi experiments and incubations with pathway inhibitors/activators

For depletion experiments, siRNA oligonucleotides (20 nmol/l) were introduced to the cells by electroporation (1,300 V, 20 ms, 2 pulses) using the Neon Transfection system at day eight of differentiation. Incubations with inducers/inhibitors of the UPR (tunicamycin [5 μg/ml], 4μ8C [64 μmol/l], ceapin-A7 [4.7 μmol/l], GSK2656157 [5 μmol/l]), metabolic pathways and/or DNMT inhibitors (2-deoxy glucose [0.1 mmol/l], UK5099 [10 μmol/l], oligomycin [1 μmol/l], etomoxir [3 μM], RG-108 [100 μmol/l], 5-azacytidine [10 μmol/l]) were performed for 3 h (metabolic flux studies), 8 h (mRNA/metabolomic studies) or 16 h (protein studies) at day 13 of differentiation. Inhibitor concentrations were selected based on results from published studies [2,[14], [15], [16], [17], [18]].

#### 2.3.5 *In vitro* transcription and mRNA-based overexpression

*CKB* and *XBP1s* mRNA templates were synthesized from cDNA, as described below, using T7-containing primers. The resulting amplicons were isolated from agarose gels. Synthesis of mRNA was performed using the HiScribe T7 ARCA mRNA Kit (with tailing) according to the manufacturer's instructions. N1-Methylpseudo-UTP was incorporated into the mRNAs to increase stability. DNMT3A-dCas9 mRNA was generated by amplifying the DNMT3A-dCas9 cassette from a pCDNA3-DNMT3A-dCas9 plasmid. As described above for siRNAs, the in vitro synthesized mRNAs (*CKB* [20 pmol], *XBP1s* [40 pmol] and dCas9-DNMT3A [10 pmol]/1 × 10^6^ cells) were introduced to the cells at day eight of differentiation using the Neon Transfection System with the following settings (1700 V, 20 ms, one pulse).

#### 2.3.6 Generation of cells for luciferase assays

A 942-base pair long region of the *CKB* promoter was PCR amplified from purified genomic DNA with primers containing SbfI and NehI restriction enzyme sequences. Following purification and digestion as below described, the amplicon was subcloned into pLX307 expressing firefly luciferase, used to generate a stable expressing cell line. In brief, stable competent cells were transformed with ligated plasmid following manufacturer instructions and single colonies were picked and cultured. From these, plasmids were extracted using QIAprep Spin Miniprep Kit and verified by Sanger sequencing. Lentiviruses were generated by transfecting HEK293 cells with plasmids along with two packaging vectors using Opti-MEM and Lipofectamine 3000 according to the manufacturer's instructions. Viruses were harvested from conditioned media two days after transfection, filtered and quantified using Lenti-X GoStix Plus. Around 200,000 proliferating adipocytes/well were seeded without antibiotics, then spinfected with 50,000 ng of virus for 1 h, 800×*g* at 37 °C. After three days cells were selected using puromycin (1 ng/ml). The selection was carried out until non-infected control cells cultured in parallel, were no longer viable [19]. Once cells were selected and expanded, they were plated into 96 well plate format with a density of 12 × 10^6^/well. At day 13 of differentiation, cells were treated with the indicated drugs as explained in the previous paragraphs and luciferase levels were quantified using the Pierce Firefly Luciferase Glow Assay Kit according to the manufacturer's instructions. All measurements were adjusted to the respective sample's protein concentration.

### 2.4 CellROX assay

Cells incubated with menadione were cultured with media containing CellROX dye at a final concentration of 5 μmol/l for 30 min at 37 °C. Cells were subsequently washed three times in PBS and fixed with 4% paraformaldehyde for 15 min. Three additional washes in PBS were then performed before incubation with PBS/Hoechst 1:5000 for 10 min. Finally, cells were washed three more times and incubated in PBS before reading intensity of both dyes. CellROX intensity was normalized over Hoechst. Dye intensities were quantified using a Varioskan LUX (Thermo Fisher).

### 2.5 Creatine kinase activity assay

Cells were incubated with or without tunicamycin (5 ng/ml) for 16 h. In parallel, cells were also depleted of *CKB* using siRNAs as positive control. Cells were then lysed with 200 μl homogenization buffer (HB buffer, sucrose [250 mmol/l], Tris-HCl [pH 7.4] [1 mmol/l], EDTA [pH 8] [1 mmol/l], 2% BSA). After lysis, cells were passed through a 23G needle for a total of 20 times, followed by centrifugation at 20,000×*g* for 40 min at 4 °C. The supernatant was collected, and 10 μl of the cell lysate was used to measure creatine kinase activity according to manufacturer's instructions. In parallel, protein concentration of the respective samples was quantified for normalization. To correct for background signal, the lowest value detected in si*CKB* cells was subtracted from all other values.

### 2.6 Genomic DNA isolation

Genomic DNA was purified from adipocytes using DNeasy Blood & Tissue Kit following the manufacturer's instructions and concentrations were quantified using Nanodrop 2000. Samples were then utilized for specific downstream analysis.

### 2.7 PCR, agarose gel and amplicon purification

PCR amplification was carried out using Q5 Hot Start DNA Polymerase according to manufacturer's instructions. Amplification conditions were adjusted based on primer design and amplicon length; the reaction was then performed using a Peltier Thermal Cycler PTC-225 (MJ Research). Upon completion, PCR products were run on a 0.6% agarose gel containing 1xTAE buffer (Tris [40 mmol/l], acetic acid [20 mmol/l], EDTA [1 mmol/l]) and SYBR safe DNA Gel Stain to confirm successful amplification and to excise the correct product in case of multiple bands. Purification was then performed using NucleoSpin Gel and PCR Clean-up according to the manufacturer's instructions. To display XBP1s splicing following PCR amplification of cDNA, the products were run on a 3% agarose gel containing 1xTAE and SYBR safe DNA Gel Stain to ensure proper separation of small amplicons.

### 2.8 RNA isolation, cDNA synthesis and real-time qPCR

Total RNA was extracted from human adipocytes using NucleoSpin RNA kit as previously described [2]. Concentrations and purity of RNA were quantified using Nanodrop 2000. The isolated RNA was reverse transcribed with iScript complementary DNA synthesis kit and quantification of mRNA levels were performed using SYBR-Green assays. Relative expression levels were calculated with the comparative Ct-method (2^ΔCt-target gene^/2^ΔCt-reference gene^). To quantify the levels of *XBP1*, primers around the splicing site were designed to allow detection of both spliced (*XBP1s*) and unspliced (*XBP1u*) variants, as indicated in the agarose gels.

### 2.9 Determination of CCL2 protein secretion

CCL2 secretion from conditioned culture media was measured using the MCP-1/CCL2 Human Uncoated ELISA Kit following the manufacturer's instructions. All the data generated were normalized by the total RNA concentration of the sample from where the culture media was taken.

### 2.10 Determination of adiponectin secretion

Adiponectin secretion from conditioned culture media was measured using the Human Total adiponectin/Acrp30 ELISA Kit - Quantikine following the manufacturer's instructions. All the data generated were normalized by the total Hoechst intensity of the sample from where the culture media was taken.

### 2.11 Protein extraction, quantification, gel electrophoresis and western blotting

Cells plated in six-well plates were lysed at day 13 of differentiation using RIPA buffer supplemented with a protease inhibitor cocktail and phosphatase inhibitor. Lysates were then centrifuged, phase separated and quantified with Bicinchonic acid assay per the manufacturer's instruction. Samples were diluted to normalize volume and concentration, followed by denaturation at 95 °C for 5 min with the addition of 4× Laemmli and Dithiothreitol (50 mmol/l). Proteins were loaded onto an SDS-PAGE gel, separated by electrophoresis, and transferred on PVDF membranes. The latter were blocked with 3% Blotto Immunoanalytical Grade in TBS-T (Tris [0.05 mol/l], NaCl [0.15 mol/l], pH 7,6, 0.1% Tween 20) for 1 h, followed by an overnight incubation with primary antibodies in blocking solution. The next day, membranes were washed three times 10 min each in TBS-T and then incubated with blocking solution containing a horseradish peroxidase (HRP)-conjugated secondary antibody for 1 h at room temperature. Finally, membranes were washed three more times for 10 min in TBS-T, incubated with ECL Prime Western Blotting Detection Reagent, and imaged using Chemidoc MP Imaging System.

### 2.12 Zinc staining

In brief, after protein separation on SDS-PAGE, gels were stained with E-Zinc Reversible Stain kit, according to manufacturer's instructions.

### 2.13 Seahorse metabolic flux assay and CyQUANT analysis

Real time quantification of oxygen/extracellular acidification ratio were performed using Seahorse XF96 Extracellular Flux Analyzer as previously described [2]. Briefly, cells were seeded and differentiated in Seahorse plates and, at day 13 post adipogenesis induction, adipocytes were incubated in Seahorse DMEM medium (pH 7.4) supplemented with sodium pyruvate (1 mmol/l), glutamine (2 mmol/l) and glucose (10 mmol/l). The assay was performed by sequential addition of oligomycin (inhibitor of ATP synthesis, [1.5 μmol/l]), carbonyl cyanide-p-trifluoromethoxy phenylhydrazone (FCCP, [1.5 μmol/l]) and rotenone/antimycin A (inhibitors of complex I and complex III of the respiratory chain, respectively, [0.5 μmol/l]). Seahorse data were then normalized using the CyQUANT Kit following manufacturer's instructions. In brief, immediately after the Seahorse assay, cells were incubated with the CyQUANT reagent mix and fluorescence was quantified using Varioskan LUX (Thermo Fisher) according to manufacturer instructions.

### 2.14 RNA sequencing and library preparation

Total RNA from cells incubated with or without Tm was isolated as described above and used for library preparation. The yield and quality of the amplified libraries were analyzed using Qubit (Thermo Fisher) and Tapestation (Agilent) and equal amounts were subsequently combined. Pooled samples were sequenced on the Illumina Nextseq 2000 P2 100 cycle sequencing run, generating 59 base paired end reads with dual index. Basecalling and demultiplexing was performed using CASAVA, to generate Fastq files which were aligned to GRCh38 for further downstream analysis.

### 2.15 Affinity-based purification assays

For the following assays, adipocytes seeded in 15 cm dishes (one dish per assay) were used as starting material.

#### 2.15.1 Nuclei isolation and immunoprecipitation

All solution used in the following protocol were supplemented with PIC/PI. Adipocytes were washed twice in ice-cold PBS and incubated for 15 min in 4 ml of Solution A (Tris-HCl [10 mmol/l], pH 7,5; NaCl [10 mmol/l]; MgCl_2_ [3 mmol/l]; KCl_2_ [10 mmol/l]; 0.05 % NP-40) and then collected in 50 ml tubes. Cells were lysed by passing them through a 23G needle three times. Immediately afterwards, Solution B (Tris-HCl [10 mmol/l], pH 7.5; NaCl [10 mmol/l]; MgCl_2_ [3 mmol/l]; KCl_2_ [10 mmol/l]; 0.05% NP-40; sucrose [0.6 mol/l]) in a 1:1 ratio (v/v) upon which the lysate was mixed vigorously. Following centrifugation at 500×*g* for 20 min, the supernatant was discarded and the pellet containing nuclei was resuspended in nuclear extraction buffer (HEPES [20 mmol/l], pH 7.4; NaCl [0.5 mol/l], MgCl_2_ [2 mmol/l]; CaCl_2_ [1 mmol/l]; 0.5% NP-40; KCH_2_CH_3_ [110 mmol/l]; ZnCl_2_ [1 μmol/l]). Additionally, 2 μl/ml Benzonase were added to nuclear extraction buffer only when protein immunoprecipitations were performed. The samples were subsequently rotated for 30 min at 4 °C and centrifuged at 20,0000×*g* for 10 min. To obtain nuclear proteins, the supernatant was collected and diluted with Nuclear Dilution Buffer (HEPES [20 mmol/l], pH 7.9; EDTA [1 mmol/l], 0.2% NP-40). At this step, a small aliquot was put aside as input. Indicated antibodies (3 μg per reaction) were added to the remaining samples, which were subsequently rotated overnight at 4 °C. Magnetic beads coupled with recombinant Protein A and G (50 μl of each type per reaction) were calibrated according to manufacturer's instructions and subsequently mixed with the antibody/sample solution. Following rotation for 3 h at 4 °C, a magnetic rack was used to separate the beads from the flow-through, which was collected. Five wash steps were performed in 1 ml wash buffer (Tris-HCl [10 mmol/l], pH 7.5; NaCl [150 mmol/l]; EDTA [0.5 mmol/l]; 0.05% NP-40). The beads were resuspended in PBS and transferred to a new tube where the PBS was replaced by a RIPA/Laemmli mixture supplemented with a protease inhibitor cocktail and phosphatase inhibitor. Proteins were eluted by denaturation at 95 °C for 10 min along with input and flow through samples for western blotting analysis.

#### 2.15.2 DNA affinity binding assay

Nuclear proteins were isolated as described above. A DNA template corresponding approximately to the first thousand bases of the *CKB* promoter was PCR amplified using primers with or without biotin labelling at their 5’ ends. The purity and size of the amplicon was confirmed by running one minor part of the sample on an agarose gel. Subsequently, 4 mg nuclear proteins were rotated with 1 μg of PCR product overnight at 4 °C. The following day, 100 μl of Dynal MyOne Dynabeads Streptavidin C1 beads per sample were calibrated following manufacturer's instruction and subsequently added to the oligos/sample solution. Following rotation for 3 h at 4 °C, a magnetic rack was used to separate beads from flow-through, which was collected. A total of five washes were performed using 1 ml wash buffer. Finally, beads were resuspended in PBS, transferred to a new tube where PBS was replaced by RIPA/Laemmli mixture supplemented with a protease inhibitor cocktail, phosphatase inhibitor and a reducing agent (DTT [50 μmol/l]). Proteins were eluted by denaturation at 95 °C for 10 min along with input and flow through samples for western blotting analysis.

#### 2.15.3 Methylated DNA immunoprecipitation

Adipocytes were washed twice in cold PBS and genomic DNA was extracted as described above. Thereafter, 6 μg of genomic DNA in 300 μl of TE buffer was sonicated (20 cycles, 30 s on, 30 s off) in the presence of ceramic beads in 1.5 ml Bioruptor Pico Tubes using a Bioruptor Pico sonication device to obtain DNA fragments of 300–600 bp. Quality of the procedure was confirmed by loading a small part of the sheared genomic DNA onto 2% agarose gels. One third of the genomic DNA was saved as input. The rest was hybridized at 95 °C for 10 min and immediately after incubated on ice for 5 min. The sample volume was then adjusted to 500 μl by adding 100 μl of 5× IP buffer (sodium phosphate [50 mmol/l], pH 7.0; NaCl [0.7 mol/l]; 0.25% Triton-X100) and 200 μl TE buffer. Samples were rotated overnight at 4 °C with 2 μg of anti-5-Methyl-Cytidine antibody. Magnetic beads coupled with recombinant Protein A and G (50 μl of each type per reaction) were calibrated according to manufacturer's instructions and subsequently mixed with the antibody/sample solution. Following rotation for 3 h at 4 °C, a magnetic rack was used to separate the beads and washed them three times with 1× IP buffer, incubating them each time for 5 min on rotating platform at room temperature. Thereafter, beads were resuspended with digestion buffer (Tris-HCl [50 mmol/l], pH 8.0; EDTA [10 mmol/l]; 0.5% SDS), containing 7 μl of Proteinase K (10 mg/ml), and incubated overnight on thermomixer at 55 °C degrees and 1400 rpm. Finally, precipitated DNA was purified and concentrated with NucleoSpin Gel and PCR Clean-up kit following manufacturer's instructions. Pull down enrichment was measured via real-time qPCR. Relative enrichment was calculated adjusting input for dilution factor and then obtaining the percentage of relative pull-down enrichment with the following formula 100 × 2ˆ[Adjusted Input – Ct (IP)].

#### 2.15.4 Chromatin immunoprecipitation

Chromatin immunoprecipitation was carried out with the Magna ChIP HiSens Chromatin Immunoprecipitation Kit using an anti-XBP1s antibody per the manufacturer's instructions. Pull down enrichment was measured by qPCR and was calculated as mentioned in the previous paragraph.

### 2.16 Bioinformatic analysis

Bioinformatic analyses were performed using Graph Pad Prism 9 and R studio v.4.1.1. Differential expression analysis of RNAseq data were carried out converting raw counts into cpm (count per milion), and statistical analysis using log2 cpm values to calculate both fold change and statistical significance. Gene set enrichment analysis (GSEA) for Mac (v.4.3.3) was used for pathway analysis, enriching for GO: Biological Process related pathways. Significantly enriched pathways were then plotted using ggplot2 in R studio. Principal component analysis was calculated in R studio, and ellipses generated using Euclidean model, results were then plotted using ggplot2 package. Transcriptome comparison with publicly available dataset was carried out using data from the following GEO identification numbers GSE117042 and GSE200742. Intersection of commonly significantly ER stress regulated genes was done using UpSetR package in R studio, sorting out common up- and downregulated gene annotations and plotting them with Graph Pad and pheatmap package (R studio) for bar chart and heatmaps respectively. Data related to *Ern1* adipocyte-specific knockout mice were obtained from publicly available data at the following GEO identification number, GSE180524. All the correlations involving clinical cohorts were carried out using R studio with ppcor package. Where indicated, variables were adjusted for BMI to determine the influence independently of body weight.

## 3 Results

### 3.1 *CKB* expression is suppressed by the unfolded protein response

**Figure 1:**
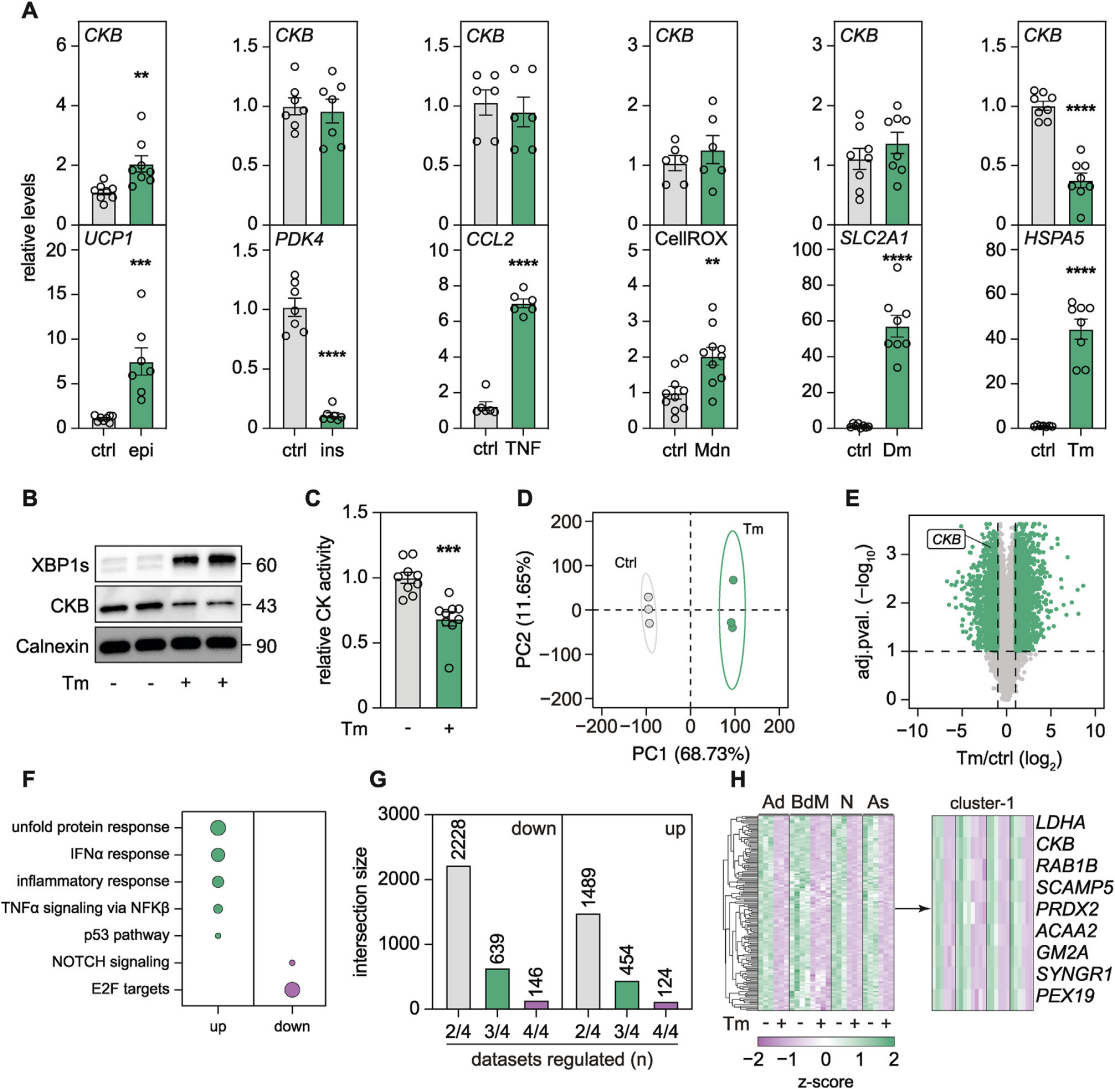
**The transcription of *CKB* is suppressed by the unfolded protein response. A.***CKB* expression (upper panels) and relevant positive controls (lower panels) for the indicated stimuli. Values are mean ± SEM. *P* values were calculated by Student's two-tailed t-test assuming unequal variance. Legends are the following: control (ctrl), epinephrine (epi), insulin (ins), tumor necrosis factor α (TNF), menadione (Mdn), dimethyloxalylglycine (Dm), tunicamycin (Tm). **B.** Western blots displaying XBP1s, CKB, and Calnexin (ER marker), from cells incubated with or without Tm. Representative blots from one out of three independent experiments. **C.** Representative creatine kinase activity of cells incubated with or without Tm. Values are mean ± SEM. *P* value was calculated by Student's two-tailed t-test assuming unequal variance. **D.** Dimension 1 and 2 from principal component analysis of 6 RNAseq samples of cells incubated with or without Tm (Ctrl = 3, Tm = 3). The ellipses display 95% confidence intervals. **E.** Volcano plot displaying differentially expressed genes of cells treated with or without Tm. **F.** Gene set enrichment analysis of significantly enriched pathways in transcriptomic data from adipocytes incubated with or without Tm. Plotted are the pathways enriched by *P* < 0.05, dot size is proportional to the Normalized Enrichment Score. **G.** Differentially up- and downregulated genes across multiple datasets of cells incubated with or without Tm. **H.** Heatmap of differentially downregulated genes across all datasets analyzed with focus on the cluster including *CKB* (Ad = Adipocytes, BdM = Bone marrow derived Macrophages, N = Neurons, As = Astrocytes). Statistical significance is presented as following: *P* < 0.05 ∗, *P* < 0.01 ∗∗, *P* < 0.001 ∗∗∗, *P* < 0.0001 ∗∗∗∗.

To dissect mechanisms governing *CKB* expression in human white adipocytes, we screened a wide array of conditions known to impact on fat cell function in health and disease. As a positive control, we included an adrenoceptor agonist (epinephrine) and confirmed that this treatment increased *CKB* mRNA levels, thereby extending recent findings in mouse brown adipocytes to white fat cells [3] (Figure 1A). Stimulations with insulin, tumor necrosis factor alpha, menadione (a reactive oxygen species inducer) and dimethyloxalylglycine (a hypoxia-mimetic agent) did not affect *CKB* expression (Figure 1A). In contrast, we found that the ER stress inducer, tunicamycin (Tm), resulted in a pronounced reduction in both CKB mRNA and protein levels as well as attenuated creatine kinase activity (Figure 1A–C). Furthermore, incubation with palmitate, an established ER stress inducer [20,21], resulted in a similar reduction of *CKB* expression (Figure S1). We next tested the universality of our findings by generating RNA sequencing data from white adipocytes incubated in the presence or absence of Tm and compared the results with publicly available transcriptomic datasets from Tm-treated bone marrow-derived macrophages [22], primary neurons and astrocytes [23] (Table S3). We found that **i**) ER stress induction resulted in a major transcriptional rewiring of white adipocytes including induction of inflammatory pathways (Figure 1D–F), **ii**) 270 “core” genes were congruently altered by Tm treatment across all four cell types (Figure 1G), and **iii**) *CKB* was one of the top down-regulated genes in all datasets (Figure 1H). Taken together, these findings identify *CKB* repression following ER stress induction as a transcriptional response that is conserved across multiple cell types.

### 3.2 *CKB* mRNA levels are reduced by the IRE1–XBP1s axis

**Figure 2:**
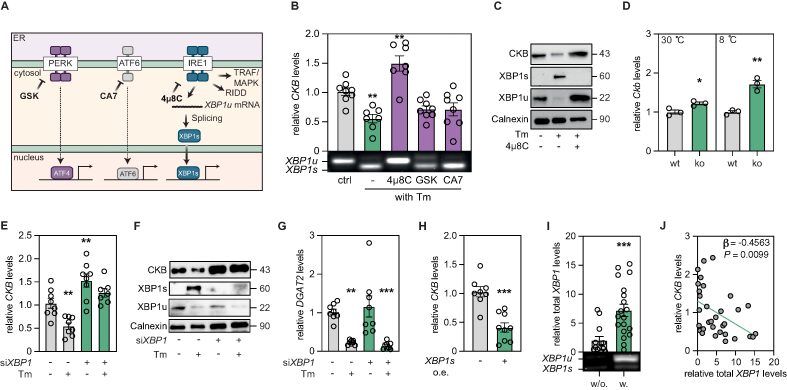
**The unfolded protein response mediates *CKB* downregulation via the IRE1–XBP1s axis. A.** Graphical representation of the ER stress branches, focusing on the IRE1-XBP1s axis. **B.***CKB* expression in adipocytes incubated with or without Tm, and 4μ8C, GSK or CA7. The total XBP1 qPCR product was loaded on agarose gels to confirm splicing and results are displayed underneath the bar plot. Values are mean ± SEM. Statistical significance was calculated using one-way ANOVA comparing all the conditions to control. **C.** Western blots for XBP1s, XBP1u, CKB, and Calnexin (ER marker), from adipocytes incubated with or without Tm and 4μ8C. Representative example based on three samples from one out of three independent experiments. **D.***Ckb* expression in inguinal white adipose tissue of *WT*/*Ern1*^*Adipo-Cre*^ mice fed a high fat diet for 16 weeks (n = 3 each group), housed at either thermoneutrality or 8 °C. Data were retrieved from GSE180524. Values are mean ± SEM. *P* values were calculated using Student's two-tailed t-test assuming unequal variance. **E.** Gene expression of *CKB* from si*C* or si*XBP1* adipocytes, incubated with or without Tm. Values are mean ± SEM. Statistical significance was calculated using one-way ANOVA comparing all the conditions to control. **F.** Western blots for XBP1s, XBP1u, CKB, and Calnexin (ER marker), from si*C*/si*XBP1* transfected adipocytes, incubated with or without Tm. One representative example from three independent experiments. **G.***DGAT2* expression from si*C*/si*XBP1* transfected cells, incubated with or without Tm. Values are mean ± SEM. Statistical significance was calculated using one-way ANOVA comparing all the conditions to control. **H.***CKB* expression in in adipocytes transfected with mRNA encoding *XBP1s*. Values are mean ± SEM. *P* value calculated by Student's two-tailed t-test assuming unequal variance. **I.** Gene expression of total *XBP1* in human white adipose tissue of subjects with (w., n = 19) or without (w/o, n = 20) obesity. Agarose gel displaying splicing of *XBP1*, is shown underneath the plot. Values are mean ± SEM. *P* value calculated by Student's two-tailed t-test assuming unequal variance. **J.** Spearman correlation of *CKB* vs. total *XBP1* gene expression in adipose tissue of women with (n = 19) or without (n = 20), obesity. β and *P* values from multiple regression analysis using BMI as a co-variate are displayed. Statistical significance is presented as following: *P* < 0.05 ∗, *P* < 0.01 ∗∗, *P* < 0.001 ∗∗∗, *P* < 0.0001 ∗∗∗∗.

ER stress occurs when the protein-folding capacity of the ER is overwhelmed [24,25]. In response to this, cells activate a highly conserved adaptive pathway known as the unfolded protein response (UPR), which facilitates restoration of ER homeostasis [24,25] via three integral membrane proteins termed protein kinase RNA-like ER kinase (PERK), activating transcription factor 6 (ATF6), and inositol requiring enzyme 1 (IRE1) [[24], [25], [26]]. These sensors detect when the protein folding capacity in the ER is exceeded and relay this information to the nucleus via transcription factors (Figure 2A). To define which UPR branch regulates *CKB* expression, we incubated the cells with Tm in the presence or absence of pathway-selective inhibitors. Tm-treatment promoted expression of the general ER-stress response gene *HSPA5*, and this was significantly reduced whenever cells were co-treated with one of the UPR branch-selective inhibitors (Figure S2A). In contrast, we found that while PERK and ATF6 inhibition (using GSK256167 [GSK]and ceapin-A7 [CA7], respectively) did not impact on *CKB* mRNA levels, treatment with the IRE1 inhibitor 4μ8C abrogated the effects of Tm on both CKB mRNA expression and protein abundance (Figure 2B–C, Figure S2B). To exclude possible crosstalk between the UPR, we tested double-inhibition upon ER stress induction of UPR branches, resulting in HSPA5 levels indistinguishable from control cells. In contrast, while CKB is downregulated by Tm, this effect is only reversed in the presence of the IRE1-XBP1s inhibitor (Figures S2B). These findings were reproduced in murine 3T3-L1 adipocytes (Figure S2C) and extended in vivo by mining transcriptomic data from a recent study where IRE1 (encoded by *Ern1*) was specifically deleted in mouse adipocytes [27]. For the latter, we found that the knockout animals exhibited higher expression of *Ckb* in their inguinal adipose tissue compared to control littermates, particularly upon cold exposure (Figure 2D). This could be mediated via the ADRA1A-Gq pathway, which is activated upon cold exposed mice as recently shown [3].

As displayed in Figure 2A, IRE1 is a serine–threonine kinase and endoribonuclease that regulates ER protein load via three distinct mechanisms. These include **i)** mRNA degradation via regulated IRE1-dependent decay (RIDD), **ii)** non-canonical activation of tumor necrosis factor receptor-associated factor (TRAF) family members, and **iii)** induction of gene transcription via RNA-splicing and activation of the transcription factor X-box-binding protein 1 (XBP1) [28]. To dissect between these regulatory circuits, we depleted cells of *XBP1*. This abrogated the Tm-induced downregulation of CKB gene and protein abundance without affecting the expression of *HSPA5* or adipocyte differentiation (Figure 2E–F and Figures S2D–F). Moreover, our observations suggest that *CKB* is not degraded by IRE1, given that known RIDD-targets such as *DGAT2* [28,29] were still regulated following *XBP1* depletion (Figure 2G). Furthermore, as shown in Figure 1A, TNFα, which activates TRAF and its respective downstream signaling pathway, did not impact on *CKB* mRNA expression. These results suggest that XBP1, but not RIDD and TRAF, mediates the UPR-induced repression of *CKB*. For the experiments described above, we observed that *CKB* expression was higher in cells where the IRE1-XBP1 branch was pharmacologically or genetically inhibited compared to control adipocytes. This suggest that *CKB* is tonically repressed by the IRE1-XBP1s branch under basal conditions, an effect which is alleviated upon inhibition of this pathway, resulting in increased *CKB* expression.

We next focused our studies on XBP1. First, we overexpressed XBP1s in human adipocytes. For this, we in vitro synthesized *XBP1s* mRNA, electroporated it into the cells and measured effects on *CKB* regulation. Compared to control adipocytes, XBP1s overexpression was evident one day post-transfection and resulted in a marked reduction of *CKB* expression with no significant effects on mature adipocytes markers (Figure 2H and Figures S2G–I). Second, we investigated the relationship between *XBP1* splicing and *CKB* expression in white adipose tissue samples from a cohort of people living with and without obesity [2]. In the individuals with obesity, we found higher abundance of both *XBP1* expression and splicing, and the total levels correlated negatively with *CKB* (rho = −0.5129; *P* = 0.0027), also after correction for BMI in a multiple regression analysis (Figure 2I–J). Altogether, our data identify the UPR as a potent suppressor of *CKB* transcription in human and murine adipocytes and we show that XBP1s is required and sufficient to mediate these effects.

### 3.3 XBP1s is recruited to the proximal promoter of *CKB*

**Figure 3:**
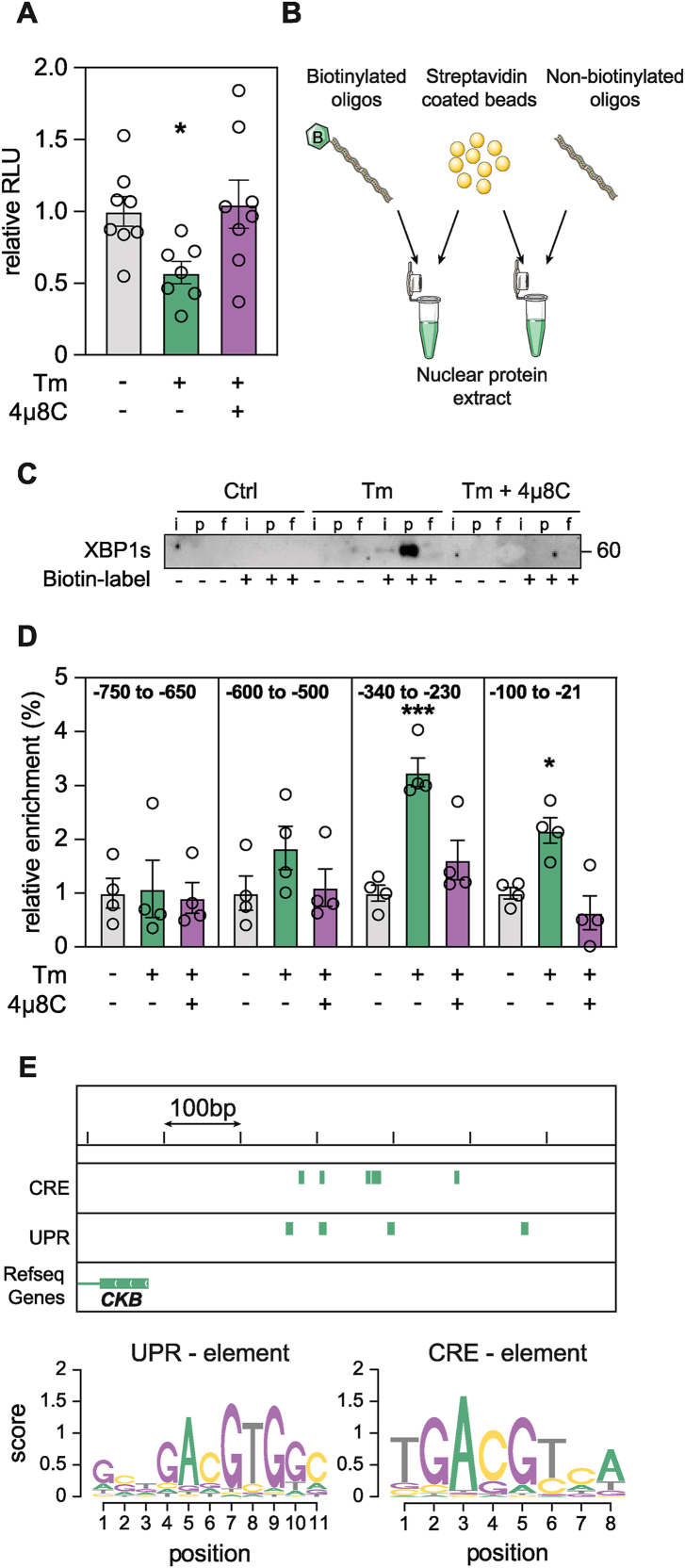
**XBP1s is recruited to the proximal promoter of *CKB.* A.** Representative example of luciferase activity expressed as relative light units in adipocytes incubated with or without Tm and 4μ8C. Values are mean ± SEM. Statistical significance was calculated using one-way ANOVA comparing all the conditions to control. **B.** Graphical representation of the DNA affinity binding assay. **C.** Representative example of the outcome of the DNA affinity binding assay. Nuclear protein lysates were isolated from adipocytes treated with or without Tm and 4μ8C. As a specificity control, primers were either biotin labeled or not as indicated. Loading labels are the following for each condition: input (i), pull down (p) and flow through (f). The image is one representative example out of three independent experiments. **D.** Representative XBP1s ChIP-qPCR on chromatin derived from cells incubated with or without Tm and 4μ8C. Primers were designed to span the indicated regions and values are mean ± SEM. Statistical significance was calculated using one-way ANOVA comparing all the conditions to control. **E.** Graphical representation of chromosome 14 focusing on the first 600 bps upstream of the *CKB* TSS. Putative XBP1s binding sites via either UPR or CRE elements are indicated by green bars and position weight matrices are displayed below. Statistical significance is presented as following: *P* < 0.05 ∗, *P* < 0.01 ∗∗, *P* < 0.001 ∗∗∗, *P* < 0.0001 ∗∗∗∗.

As XBP1s preferentially binds to proximal promoter elements [30,31], we next performed reporter and pull-down assays. For the former, we generated adipocytes stably expressing firefly luciferase under the control of the ∼1st thousand base pairs (in relation to the transcriptional start site [TSS]) of the *CKB* promoter. By incubating these cells in the presence or absence of Tm and 4μ8C, we found that the regulation of *CKB* gene expression by XBP1s occurs within this region (Figure 3A). To test if XBP1s binds to the *CKB* promoter, we next amplified a similar DNA stretch used for the luciferase assay with biotinylated primers. The resulting amplicon was thereafter purified, incubated with nuclear eluates from adipocytes treated with or without Tm and 4μ8C and pulled down using streptavidin magnetic beads (Figure 3B). As shown in Figure 3C, this revealed that the UPR increases the occupancy of XBP1s at the *CKB* promoter. To validate these findings and define the regulatory region more precisely, we performed chromatin immunoprecipitation of XBP1s followed by qPCR. By testing multiple primer sets, our data showed that XBP1s binds within the first ∼400 base pairs upstream of the *CKB* TSS in the presence of Tm, an enrichment which was abrogated by 4μ8C treatment (Figure 3D). As XBP1s binds to UPR and cAMP-responsive elements [32,33], we searched for these motifs upstream of the *CKB* TSS (Figure 3E). We identified multiple potential binding sites in the −150 to −400 base pair region, which corroborate our experimental data showing that the UPR enhances XBP1s recruitment near the proximal promoter of CKB.

### 3.4 The *CKB* promoter is methylated following induction of the UPR

**Figure 4:**
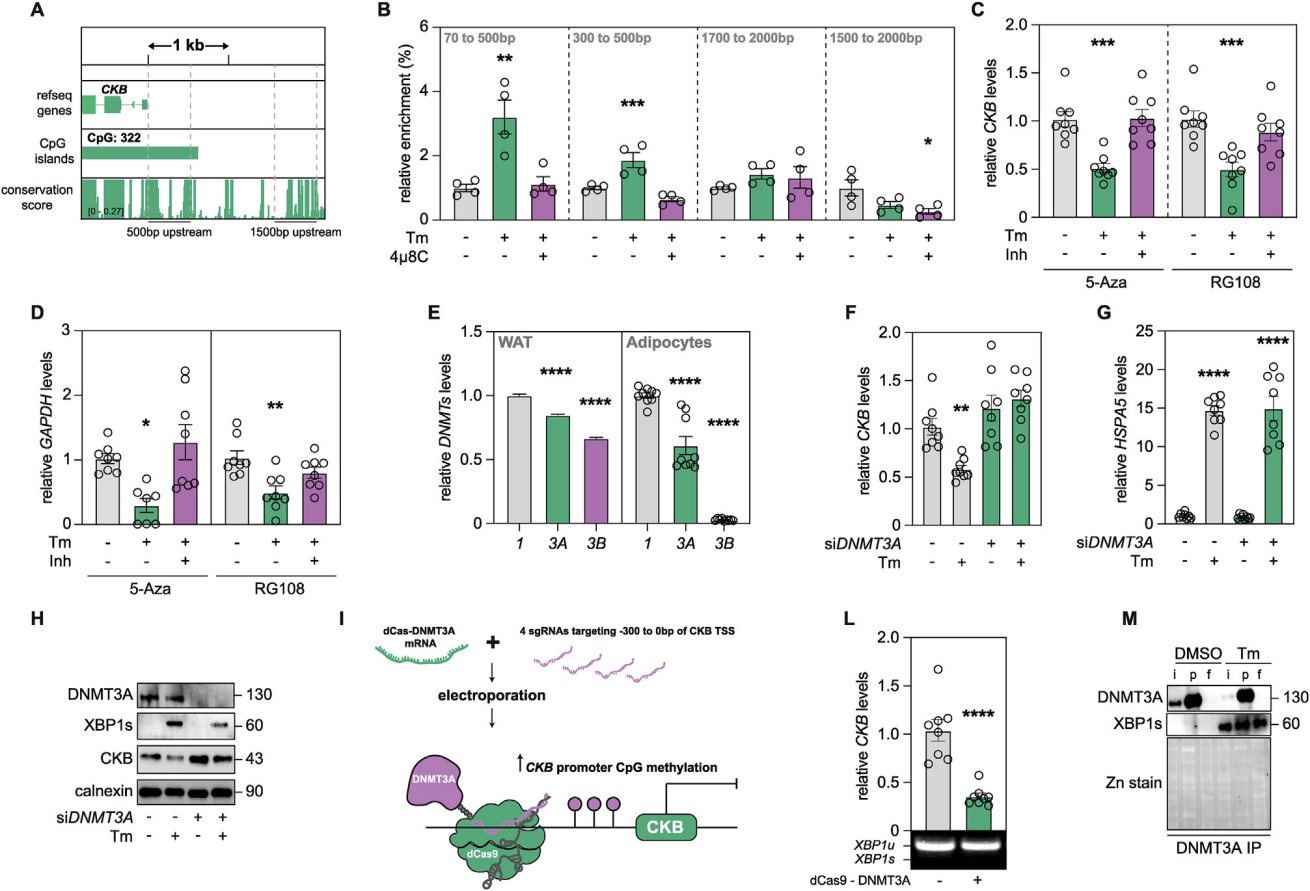
**The *CKB* promoter is hypermethylated by DNMT3A following induction of the UPR**. **A.** Graphical visualization of the genomic area of Chromosome 14 focusing on the 2000 bps upstream of the *CKB* TSS on the first row. Annotated CpG Island and sequence conservation across 20 mammal species are displayed in the rows below. **B.** Representative meDIP-qPCR, showing relative DNA methylation enrichment compared to input of adipocytes incubated with or without Tm and 4μ8C. Values are mean ± SEM. Statistical significance was calculated using one-way ANOVA comparing all the conditions to control. **C.** Gene expression of *CKB* in cells incubated with or without Tm, together with either 5-Azacytidine or RG108. Values are mean ± SEM. Statistical significance was calculated using one-way ANOVA comparing all the conditions to control. **C.** Gene expression of *CKB* in cells incubated with or without Tm, together with either 5-Azacytidine or RG108. Values are mean ± SEM. Statistical significance was calculated using one-way ANOVA comparing all the conditions to control. **D.** Gene expression of *GAPDH* in cells incubated with or without Tm, together with either 5-Azacytidine or RG108. Values are mean ± SEM. Statistical significance was calculated using one-way ANOVA comparing all the conditions to control. **E.** Gene expression of DNA methyl transferases (*DNMTs*) isoforms relative to *DNMT1*. Expression of *DNMTs* in either human white adipose (n = 16) or cultured white adipocytes are shown as indicated. Values are mean ± SEM. Statistical significance was calculated using one-way ANOVA comparing all the conditions to *DNMT1* expression. **F.** Gene expression of *CKB* from si*C* or si*DNMT3A* transfected cells, incubated with or without Tm. Values are mean ± SEM. Statistical significance was calculated using one-way ANOVA comparing all the conditions to control. **G.** Gene expression of *HSPA5* from si*C* or si*DNMT3A* transfected cells, incubated with or without Tm. Values are mean ± SEM. Statistical significance was calculate using one-way ANOVA comparing all the conditions to control. **H.** Western blots for DNMT3A, XBP1s, CKB, and Calnexin (ER marker), from si*C* or si*DNMT3A* transfected cells incubated with or without Tm. Representative example from one out of three independent experiments. **I.** Graphical representation of the dCas-DNMT3A assay. **L.** Gene expression of *CKB* in cells transfected with mRNA encoding dCas9-DNMT3A. Underneath the plot, an agarose gel loaded with the total XBP1 qPCR product is displayed. Values are mean ± SEM. *P* value was calculated by Student's two-tailed t-test assuming unequal variance. **M.** Western blots for DNMT3A immunoprecipitation from isolated nuclear proteins lysates of cells incubated with or without Tm. A Zinc stain is included underneath the western blots as a loading control of the gel. Samples were loaded as indicated: input (i), pull down (p) and flow through (f) of each sample. Representative example from two independent experiments. Statistical significance is presented as following: *P* < 0.05 ∗, *P* < 0.01 ∗∗, *P* < 0.001 ∗∗∗, *P* < 0.0001 ∗∗∗∗.

To complement our findings, we performed sequence alignments and searched for additional regulatory features proximal to the TSS of *CKB*. This revealed that well-conserved regions within the first ∼500 base pairs were located in a CpG island (Figure 4A). These regions are prone to be methylated, a dynamic process which is governed by specific enzymes that either add or remove methyl groups on cytosine bases in the DNA [34]. We hypothesized that the regulation of *CKB* by XBP1s could be mediated by changes in DNA methylation. To test this, we incubated adipocytes in the presence or absence of Tm and 4μ8C and measured CpG DNA methylation status. Through methylated DNA immunoprecipitation, we found the promoter region of *CKB* to display increased methylation levels following Tm-treatment (Figure 4B). This was reversed by pre-incubating the cells with 4μ8C and was specific for the first 500 base pair-long region of the promoter as further distal regions were not differentially methylated by any of the treatments. To assess whether these changes were required to regulate *CKB* mRNA levels upon induction of the UPR, we co-treated cells with or without Tm and two unspecific DNA methyltransferase (DNMT) inhibitors: 5-Azacytidine (5-Aza) and RG108. As a positive control, we used *GAPDH* as this gene displays methylation-dependent transcriptional regulation [35]. Our results revealed that both drugs abrogated the Tm-induced reduction in *CKB* and *GAPDH* mRNA levels (Figure 4C–D). This was specific for these genes as the Tm-mediated induction of *HSPA5* expression was unaffected by either inhibitor (Figure S3A). Therefore, our data suggest that the UPR induces methylation in the proximal promoter of *CKB*, an event that is required to repress gene transcription at this locus.

### 3.5 ER stress induces *CKB* promoter methylation via DNMT3A

While DNMT1 maintains DNA methylation, only DNMT3A-B are responsible for *de novo* CpG methylation [36,37]. We therefore measured the expression of the corresponding genes and found *DNMT3A* to be more highly expressed than *DNMT3B* in our cell model (Figure 4E). We therefore silenced *DNMT3A* and treated the cells with or without Tm. Compared to control adipocytes electroporated with scrambled duplexes, knockdown of *DNMT3A* abrogated the UPR-mediated effects on CKB mRNA and protein abundance, without affecting *HSPA5* expression and adipocyte differentiation (Figure 4F–H and Figures S3B–C). To test sufficiency, we complemented these experiments by recruiting a catalytically inactivated Cas9 fused with DNMT3A to the *CKB* promoter. This resulted in a reduction of *CKB* mRNA without effects on XBP1 splicing or adipogenesis (Figure 4I-L and Figures S3D–E). Finally, we immunoprecipitated DNMT3A from nuclear extracts of adipocytes treated with or without Tm and found an enrichment of XBP1s in the former (Figure 4M). Taken together, these findings suggest that a XBP1s–DNMT3A axis hypermethylates the proximal *CKB* promoter upon ER stress which results in repressed gene expression.

### 3.6 UPR induction results in increased glycolysis and *CCL2* expression

**Figure 5:**
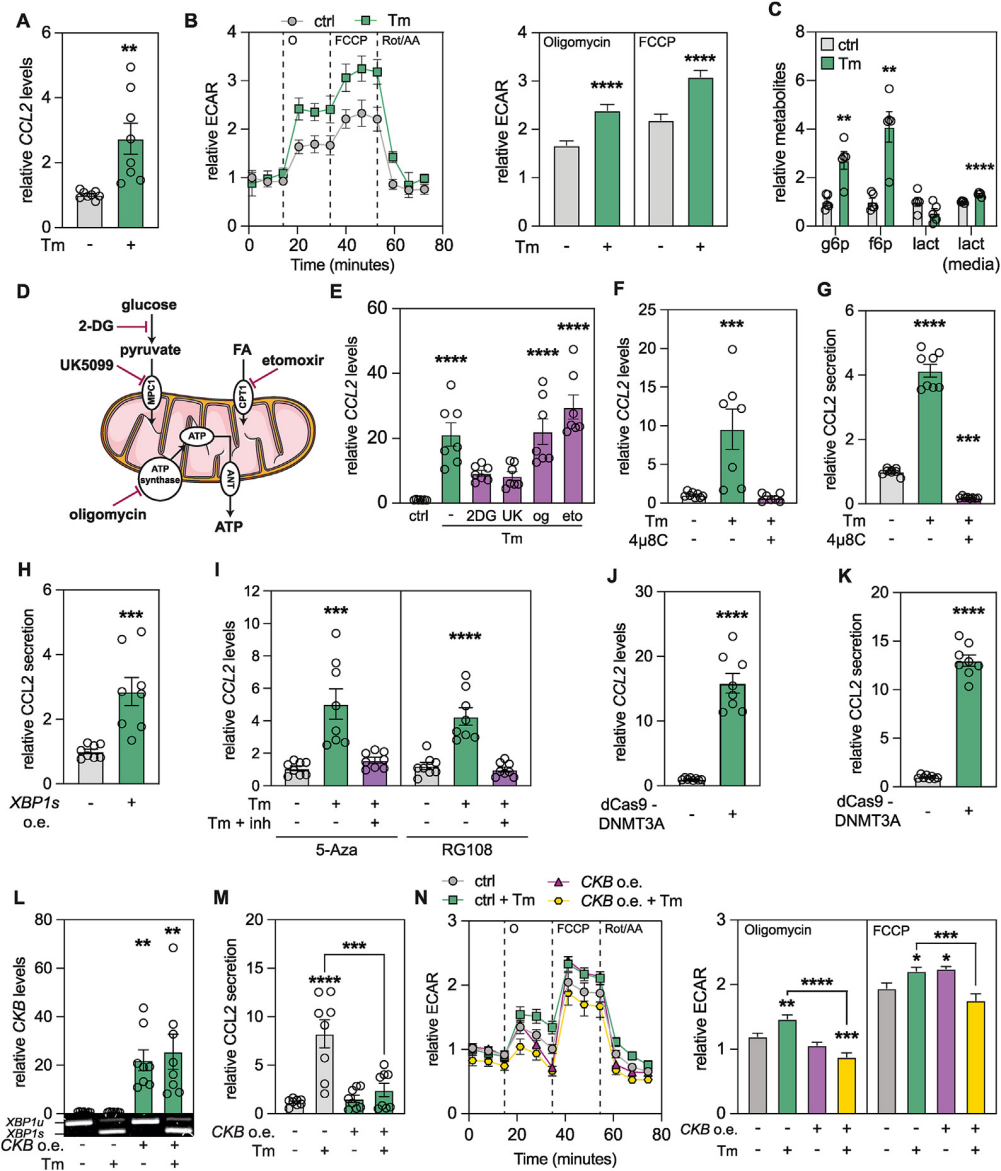
***CKB* promoter methylation and gene repression is linked to increased CCL2 secretion. A.** Gene expression of *CCL2* in cells incubated with or without Tm. Values are mean ± SEM. *P* value was calculated by Student's two-tailed t-test assuming unequal variance. **B.** Representative relative extracellular acidification rate of cells incubated with or without Tm. Values are mean ± SEM. Quantifications were performed by normalizing to the mean of the control time points before addition of oligomycin and are summarized in the right panel. Values are mean ± SEM. *P* values were calculated by Student's two-tailed t-test assuming unequal variance. **C.** Glycolysis related metabolites of cells incubated with or without Tm. Values are mean ± SEM. *P* values were calculated by Student's two-tailed t-test assuming unequal variance. **D.** Schematic representation of all the inhibitors used to selectively target intracellular metabolic pathways. **E.** Gene expression of *CCL2* in cells incubated with or without Tm together with selective inhibitors of intracellular metabolic pathways (2DG = 2-Deoxy Glucose, UK=UK5099, og = Oligomycin, eto = Etomoxir). Values are mean ± SEM. Statistical significance was calculate using one-way ANOVA comparing all the conditions to control. **F.** Gene expression of *CCL2* in cells incubated with or without Tm and 4μ8C. Values are mean ± SEM. Statistical significance was calculated using one-way ANOVA comparing all the conditions to control. **G.** Secretion of CCL2 in media harvested from cells incubated with or without Tm and 4μ8C. Values are mean ± SEM. Statistical significance was calculated using one-way ANOVA comparing all the conditions to control. **H.** Secretion of CCL2 in media harvested from cells transfected with mRNA encoding *XBP1s* after 24 h. Values are mean ± SEM. *P* value was calculated by Student's two-tailed t-test assuming unequal variance. **I.** Gene expression of *CCL2* in cells incubated with or without Tm, together with 5-Azacytidine or RG108. Values are mean ± SEM. Statistical significance was calculated using one-way ANOVA comparing all the conditions to control. **J.** Gene expression of *CCL2* in cells transfected with mRNA encoding dCas9-DNMT3A. Values are mean ± SEM. *P* value was calculated by Student's two-tailed t-test assuming unequal variance. **K.** Secretion of CCL2 in media harvested from cells transfected with mRNA encoding dCas9-DNMT3A. Values are mean ± SEM. *P* value was calculated by Student's two-tailed t-test assuming unequal variance. **L.** Gene expression of *CKB* in cells transfected with mRNA encoding *CKB* incubated with or without Tm. An agarose gel loaded with the total XBP1 qPCR product is displayed underneath the plot to show splicing upon ER stress induction. Values are mean ± SEM. Statistical significance was calculated using one-way ANOVA comparing all the conditions to control. **M.** Secretion of CCL2 into conditioned media harvested from cells transfected with mRNA encoding CKB. Values are mean ± SEM. Statistical significance was calculated using non-parametric one-way ANOVA (Geisser-Greenhouse). **N.** Representative relative extracellular acidification rate of cells transfected with mRNA encoding *CKB* incubated with or without Tm. Values are mean ± SEM. Quantification and normalization were performed as described in panel B and are summarized in the right panel. Values are mean ± SEM. Statistical significance was calculated using one-way ANOVA comparing all the conditions to control. Statistical significance is presented as following: *P* < 0.05 ∗, *P* < 0.01 ∗∗, *P* < 0.001 ∗∗∗, *P* < 0.0001 ∗∗∗∗.

In white adipocytes, *CKB* repression induces the expression of the pro-inflammatory chemokine *CCL2* via a bioenergetic switch favoring increased glycolysis [2]. To test if UPR induction phenocopies these events, we incubated adipocytes with or without Tm and measured effects on *CCL2* expression and energy metabolism using qPCR, targeted mass spectrometry and high-resolution respirometry. We found that Tm induced *CCL2* mRNA levels and increased both the extracellular acidification rate (ECAR, an indirect measure of glycolysis) and the levels of glycolytic metabolites (Figure 5A–C), without affecting intermediates in the tricarboxylic cycle or the oxygen consumption rate (Figures S4A–B). Furthermore, inhibition of glycolysis using 2-deoxyglucose or UK5099 (inhibitors of hexokinase and mitochondrial pyruvate translocation, respectively) abrogated the effects of Tm on *CCL2* expression, while oligomycin and etomoxir (inhibitors of ATP synthase and fatty acid oxidation, respectively) had no effects (Figure 5D–E). The relationship between ER stress, glycolysis and *CCL2* was not a general effect on gene expression as *HSPA5* induction by Tm was unaffected by any of the inhibitors (Figure S4C). Thus, UPR induction results in similar effects on bioenergetic pathways and *CCL2* expression as *CKB* repression alone.

### 3.7 *CKB* repression is required for ER stress-induced *CCL2* expression

To test if *CKB* promoter methylation and gene repression via XBP1s is linked to changes in CCL2 production, we measured the effects on this chemokine in our experimental settings. First, we confirmed that 4μ8C treatment reversed effects of Tm on CCL2 mRNA and protein levels (Figure 5F–G). Conversely, XBP1s overexpression induced CCL2 abundance (Figure 5H). We next investigated whether perturbing methylation events resulted in altered CCL2 abundance. This revealed that DNMT inhibition reversed the effects of Tm on *CCL2* expression (Figure 5I). Furthermore, recruitment of Cas9-DNMT3A to the *CKB* promoter increased CCL2 mRNA levels and secretion (Figure 5J–K). Thus, both gain- and loss-of-function approaches targeting XBP1s and DNMT3A resulted in reciprocal changes in CCL2 and CKB abundance. To establish if ER-stress induced repression of CKB is required for CCL2 upregulation, we overexpressed *CKB* in the presence or absence of Tm. We found that electroporation of *CKB* mRNA resulted in a pronounced increase of CKB mRNA and protein with no significant alterations in adipogenesis (Figure 5L and Figures S4D–F). While this did not affect the induction of ER stress by Tm as measured by XBP1 splicing (Figure 5L and Figure S4D), the Tm-mediated increase in CCL2 secretion and ECAR was abrogated (Figure 5M−N). Altogether, our results show that XBP1s-DNMT3A-mediated repression of *CKB* expression is crucial for the induction of *CCL2* by ER stress.

### 3.8 White adipocyte *CKB* expression and promoter methylation are altered in obesity

**Figure 6:**
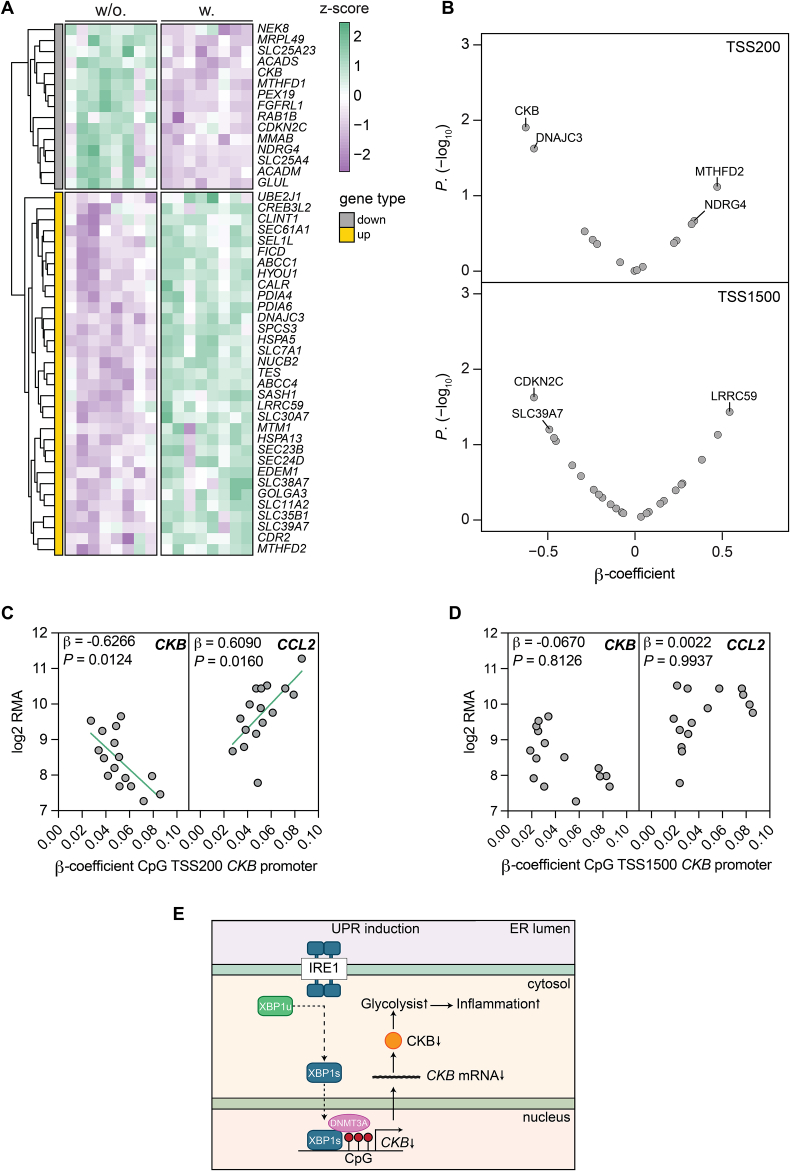
***CKB* expression and promoter methylation are altered in white adipocytes from people with obesity**. **A.** Heatmap displaying the expression of core ER stress-regulated genes (identified in Figure 1) in women with (w., n = 8) or without (w/o., n = 8) obesity. Only the significantly differentially expressed genes are plotted. Genes consistently up- or down regulated by Tm in four cell models are indicated by yellow or grey, respectively. **B.** Volcano plots displaying the Spearman's rank correlation (rho) between the methylation patterns observed at the TSS 200 and 1500 and the respective gene expression for the core ER stress-regulated genes in human adipose tissue. **C.** Spearman's rank correlation between *CKB* and *CCL2* gene expression in adipose tissue in relation to the β*-*value (CpG methylation) 200 base pairs upstream of the *CKB* TSS. Correlations remained significant even after multiple regression analysis using BMI as a co-variate (*CKB*: β = −0.6266, *P* = 0.0124; *CCL2*: β = 0.6090, *P* = 0.0160). **D.** Same analyses as in panel C but for the β*-*value 1500 base pairs upstream of the *CKB* TSS. In panels B–D, β and *P* values are shown for multiple regression analyses after BMI correction. **E.** Schematic representation of the molecular mechanism presented in this work. Statistical significance is presented as following: *P* < 0.05 ∗, *P* < 0.01 ∗∗, *P* < 0.001 ∗∗∗, *P* < 0.0001 ∗∗∗∗.

Finally, to establish whether our findings are translated in vivo, we investigated a clinical cohort where both white adipocyte methylome and adipose tissue transcriptome data were available from women living with or without obesity [6]. In this cohort, we confirmed that the obese state is characterized by ER stress as multiple of the Tm-regulated genes (defined in Figure 1G), including *CKB*, were congruently altered comparing women living with *vs*. without obesity (Figure 6A). For these genes, we correlated their promoter methylation levels (β-value) with the corresponding mRNA levels and found that methylation at TSS200, but not TSS1500, in the *CKB* promoter displayed the strongest associations after correction for BMI (Figure 6B). Finally, in concordance with our findings in vitro, there was a reciprocal association between *CKB* and *CCL2* mRNA levels and CpG methylation at *CKB* TSS200 (*CKB*: rho = −0.6618; *P* = 0.0065; *CCL2*: rho = 0.6441; *P* = 0.0085), but not TSS1500 (*CKB*: rho = −0.3786; *P* = 0.1649; *CCL2*: rho = 0.3321; *P* = 0.2264), even after correction for BMI (Figure 6C–D). In summary, these data provide further evidence that *CKB* and *CCL2* dysregulation in obesity is linked to increased CpG methylation of the proximal promoter region of *CKB* (Figure 6E).

## 4 Discussion

The interconversion of phosphocreatine and creatine is a central bioenergetic shuttle allowing rapid generation of ATP via mitochondrial and cytosolic creatine kinases [38]. Herein, we show that the UPR, through the IRE1–XBP1s branch, impacts on this pathway by downregulating *CKB* expression via promoter methylation. This epigenetic alteration leads to enhanced glucose utilization and increased secretion of the pro-inflammatory chemokine CCL2. The latter is a well-established factor produced by adipocytes that plays a central role in governing adipose tissue inflammation [39].

The UPR is an energy demanding process, which consumes ATP via for example chaperone synthesis and protein degradation [40,41]. In human white adipocytes, *CKB* depletion increases glycolysis and intracellular ATP levels [2]. It is therefore conceivable that UPR induction represses *CKB* to generate metabolic energy and thereby resolve ER stress. This mechanism appears to be a universal cellular feature given that the UPR-mediated repression of *CKB* occurs in human and mouse adipocytes as well as macrophages, neurons and astrocytes.

Mechanistically, our data in adipocytes indicate that *CKB* repression by ER stress is mediated by the IRE1-XBP1s pathway aligning well with previous data linking this branch to metabolic phenotypes [27,41,42]. Through pull-down assays, we extend these findings and show that DNMT3A interacts with XBP1s and that this complex binds to and methylates a conserved region of the *CKB* promoter containing multiple XBP1s binding motifs. Thus, our results provide an unexpected link between ER stress, epigenetic regulation of creatine metabolism and the induction of inflammation in human white adipocytes.

Our results are primarily based on assays in cultured human adipocytes treated with Tm. However, we found similar effects with palmitate. More importantly, we also observed that freshly isolated fat cells from people living with obesity recapitulate our in vitro observations as they display ER stress, lower *CKB* expression, increased methylation of the *CKB* promoter and higher levels of *CCL2* compared to non-obese individuals. Furthermore, the associations between methylation and gene expression are pronounced for a specific region close to the TSS of *CKB*. Again, this aligns well with our experimental data where Tm induces increased occupancy of XBP1s within the first 500 base pairs of the *CKB* promoter. Together with the observation that re-expression of *CKB* attenuates CCL2 production, our data in vitro and in vivo suggest that restoration of creatine metabolism might mitigate tissue inflammation in obesity and insulin resistance.

A caveat with our study is that we have not fully tested the relevance of our findings in mice. However, we observe that adipocyte-specific *Ern1* KO mice display increased *Ckb* expression in WAT. The inverse relationship between *Ern1* and *Ckb* is recapitulated when comparing the phenotypes of adipocyte-specific knockout mice. Thus, upon high fat diet, deletion of *Ern1* leads to increased white adipose tissue browning and improved metabolic phenotype, while *Ckb* knockout mice display increased predisposition to obesity and disrupted glucose homeostasis (due to impaired thermogenesis) [27,43]. Similar data have recently been reported through pharmacological inhibition of IRE1α, which ameliorates insulin resistance and glucose intolerance in mice with diet-induced obesity [44]. Again, the effects appear to be mediated via increased thermogenesis and energy expenditure, which protects against high fat diet-induced obesity. Of note, IRE1α inhibition diminishes the accumulation of obesity-induced metabolically activated and “M1-like” macrophages, resulting in reduced adipose inflammation. While these findings suggest the potential of targeting IRE1α for the therapeutic treatment of insulin resistance and obesity, they also constitute important confounding factors that make time-course studies of high fat diet and inflammation difficult to interpret in these animal models.

In conclusion, this study uncovers a pathway by which ER stress impacts adipocyte metabolism and inflammation through epigenetic regulation of *CKB*. These findings provide insights into the molecular mechanisms linking obesity, ER stress, and metabolic dysfunction, offering potential targets for therapeutic intervention in metabolic diseases.

## Data and materials availability

All data needed to evaluate the conclusions in the paper are present in the paper and/or the Supplementary Materials. All sequencing data utilized in the paper are either already published or available on Gene Expression Omnibus (GEO) and accessible through the following GEO series accession numbers: GSE24884 (Human white adipose tissue methylarray) and GSE pending upload. All raw data can be provided upon request to the reviewers and will be made publicly available upon publication of this work.

## Funding

CIMED (M.R., S.M.)

ERC-SyG SPHERES 856404 (M.R.)

European Foundation for the Study of Diabetes Future Leaders award (N.M.)

European Foundation for the Study of Diabetes Rising Star award (S.M.)

Karolinska Institutet Consolidator Programme (N.M.)

Knut & Alice Wallenberg's foundation (N.M., M.R.)

Margareta af Uggla's foundation (M.R.)

NovoNordisk Foundation MeRIAD consortium 0064142 (M.R.)

NovoNordisk Foundation NNF20OC0061149 (N.M.)

Stockholm County Council (M.R.)

Strategic Research Program in Diabetes at Karolinska Institutet (M.R., and N.M.)

Swedish Diabetes Foundation (M.R.)

Swedish Research Council (M.R. and N.M., S.M.)

## CRediT authorship contribution statement

**Gianluca Renzi:** Writing – original draft, Visualization, Methodology, Investigation, Formal analysis, Data curation. **Ivan Vlassakev:** Methodology, Investigation. **Mattias Hansen:** Methodology, Investigation, Formal analysis. **Romane Higos:** Investigation, Formal analysis. **Simon Lecoutre:** Methodology, Investigation, Formal analysis, Conceptualization. **Merve Elmastas:** Formal analysis. **Ondrej Hodek:** Methodology, Formal analysis. **Thomas Moritz:** Methodology, Formal analysis. **Lynn M. Alaeddine:** Formal analysis. **Scott Frendo–Cumbo:** Formal analysis. **Ingrid Dahlman:** Resources. **Alastair Kerr:** Methodology, Investigation, Formal analysis. **Salwan Maqdasy:** Methodology, Investigation, Funding acquisition, Formal analysis, Conceptualization. **Niklas Mejhert:** Writing – review & editing, Writing – original draft, Visualization, Supervision, Methodology, Investigation, Funding acquisition, Formal analysis, Conceptualization. **Mikael Rydén:** Writing – review & editing, Writing – original draft, Visualization, Supervision, Funding acquisition, Conceptualization.

## Declaration of competing interest

The authors declare that they have no known competing financial interests or personal relationships that could have appeared to influence the work reported in this paper.

## Data Availability

Data will be made available on request.
