# Supplementary material for: Epigenetic suppression of creatine kinase B in adipocytes links endoplasmic reticulum stress to obesity-associated inflammation
Source: Mol Metab. 2024 Dec 13;92:102082. doi: 10.1016/j.molmet.2024.102082 (PMC11731883; doi:10.1016/j.molmet.2024.102082)
Supplement: Multimedia component 2 [file mmc2.pdf]

Cohort 1 (NCT01727245)			
**Parameters**	**w.o. obesity (n = 20)**	**w. obesity (n = 19)**	**p. value**
Age (years)	55 (51-56)	46 (43-50)	0.14
Weight (kg)	68.7 (65.67-70.73)	109.4 (104.45-115.01)	<0.0001
BMI (kg/m^2)	23.45 (22.32-25.83)	39.4 (38.29-39.81)	<0.0001
Syst BP (mm Hg)	114 (108.13-123.62)	137 (131.51-139.96)	0.0017
Diast BP (mm Hg)	70.5 (67.46-73.38)	80 (76.36-83.64)	0.0024
Body fat (%)	36.8 (33.98-39.39)	50.5 (48.58-53.36)	<0.0001
P-glucose (mmol/L)	5.15 (4.96-5.45)	5.5 (5.28-5.60)	0.40
Insulin (µU/mL)	3.98 (3.36-4.76)	13.6 (11.27-15.52)	<0.0001
HOMA-IR	0.87 (0.71-1.10)	3.39 (2.77-3.79)	<0.0001
HbA1c (mmol/mol)	34 (32.54-35.29)	34 (32.19-37.46)	0.21

Supplementary Table 2: Clinical and biological parameters for subjects included in cohort1 of the study. Data are expressed in median (95% CI). P.values were calculated using Student´s t-test assuming unequal variance between the two groups. NCT number in clinicaltrials.gov is provided.
