# Supplementary figures and images for: Epigenetic suppression of creatine kinase B in adipocytes links endoplasmic reticulum stress to obesity-associated inflammation
Source: Mol Metab. 2024 Dec 13;92:102082. doi: 10.1016/j.molmet.2024.102082 (PMC11731883; doi:10.1016/j.molmet.2024.102082)

Figure 1B

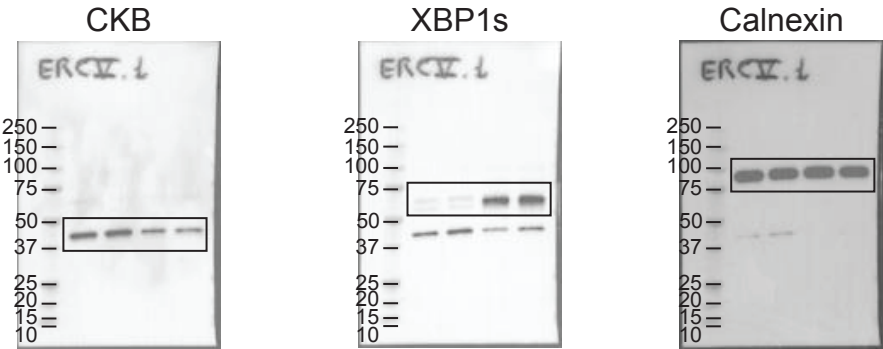

Figure 2C

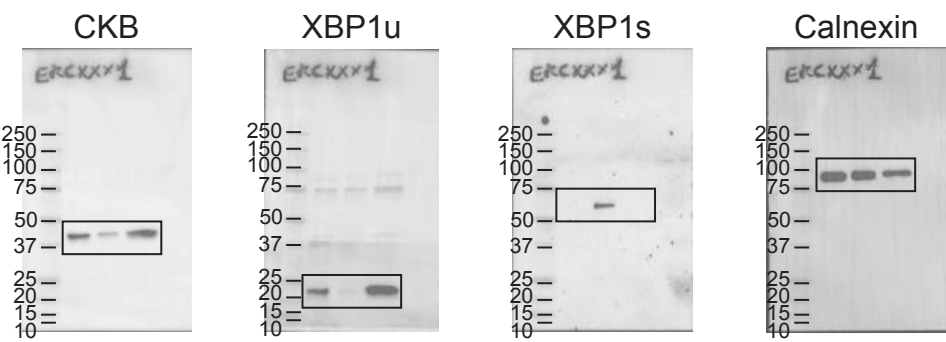

Figure 2F

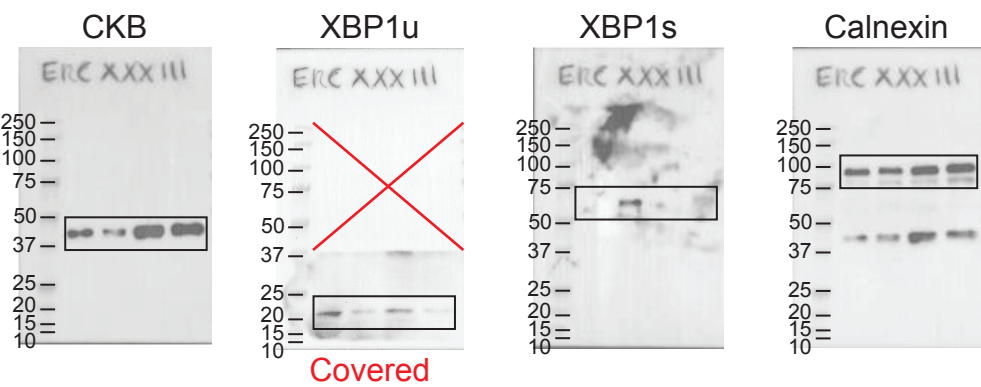

Figure 3C

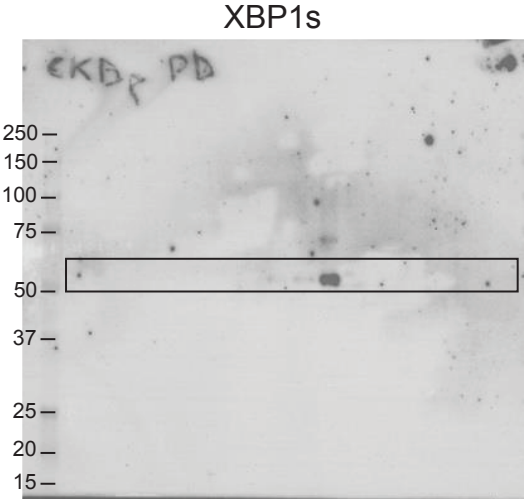

Figure 4H

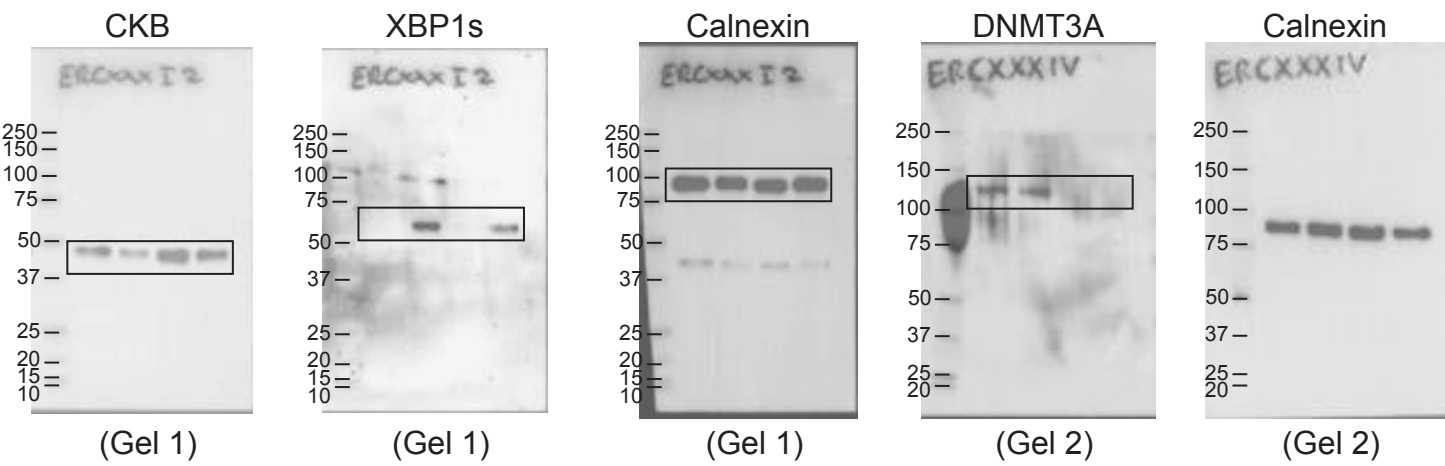

Figure 4M

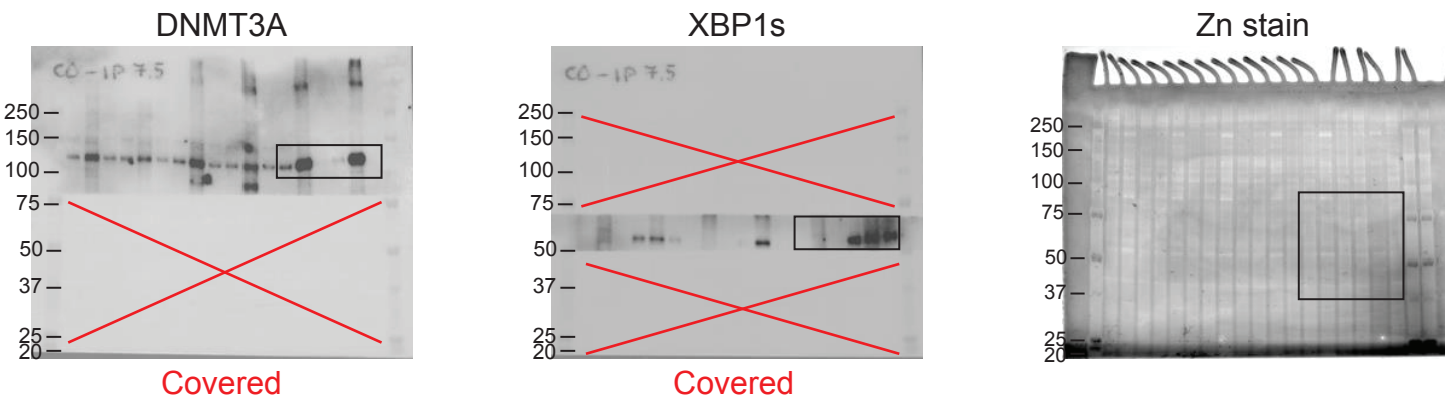

Figure S4D

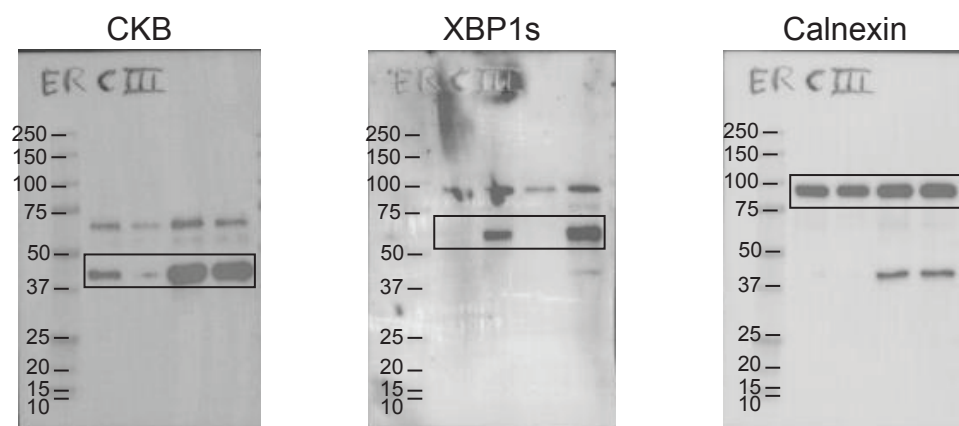

Supplement: Multimedia component 4 [file mmc4.pdf]

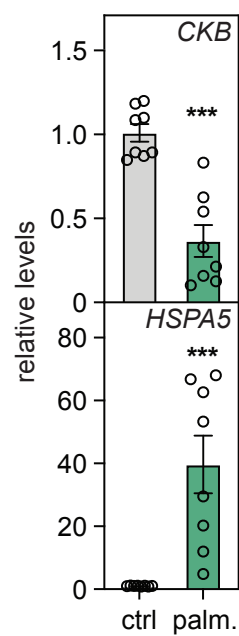

Supplement: Multimedia component 5 [file mmc5.pdf]

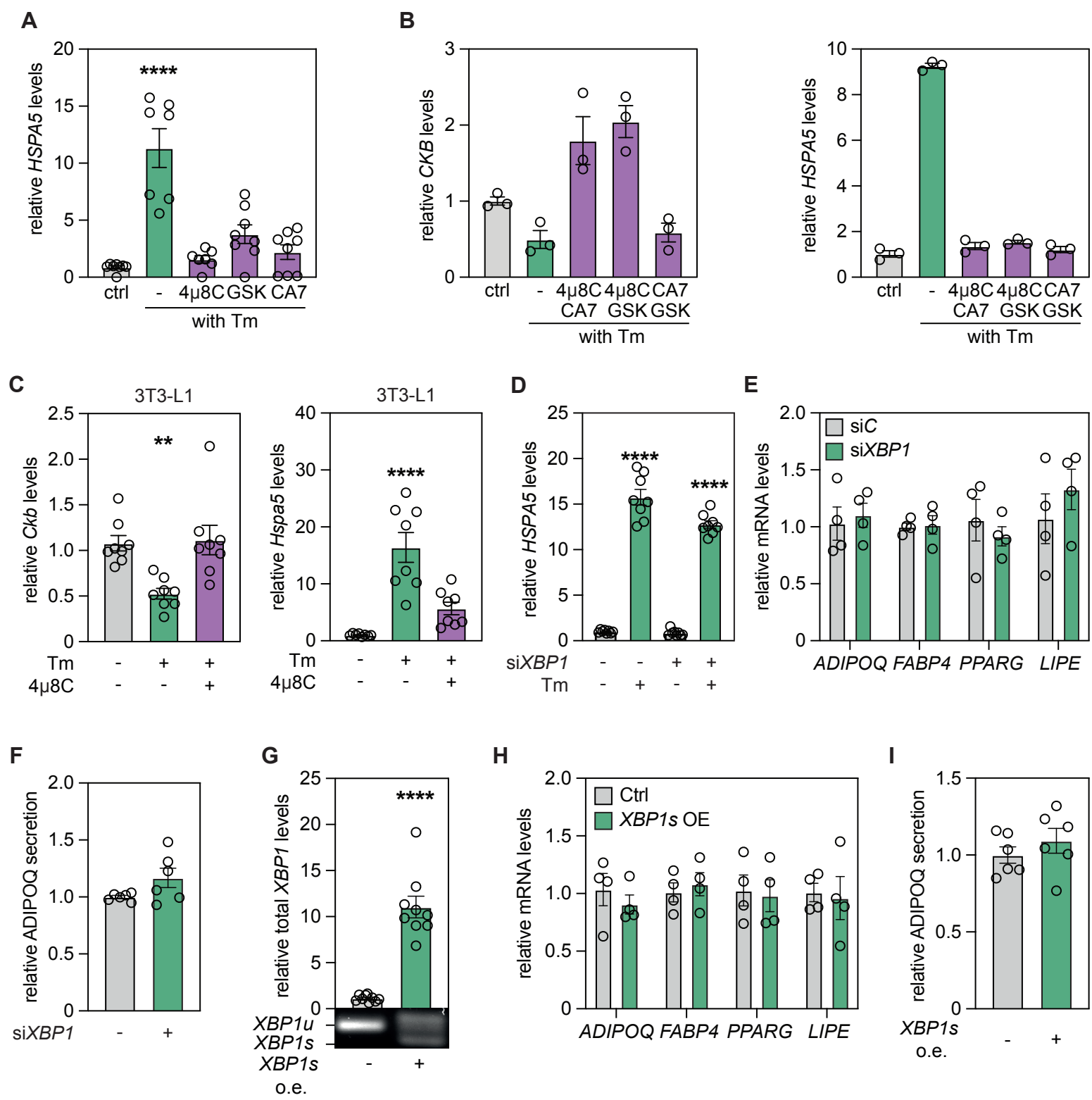

Supplement: Multimedia component 6 [file mmc6.pdf]

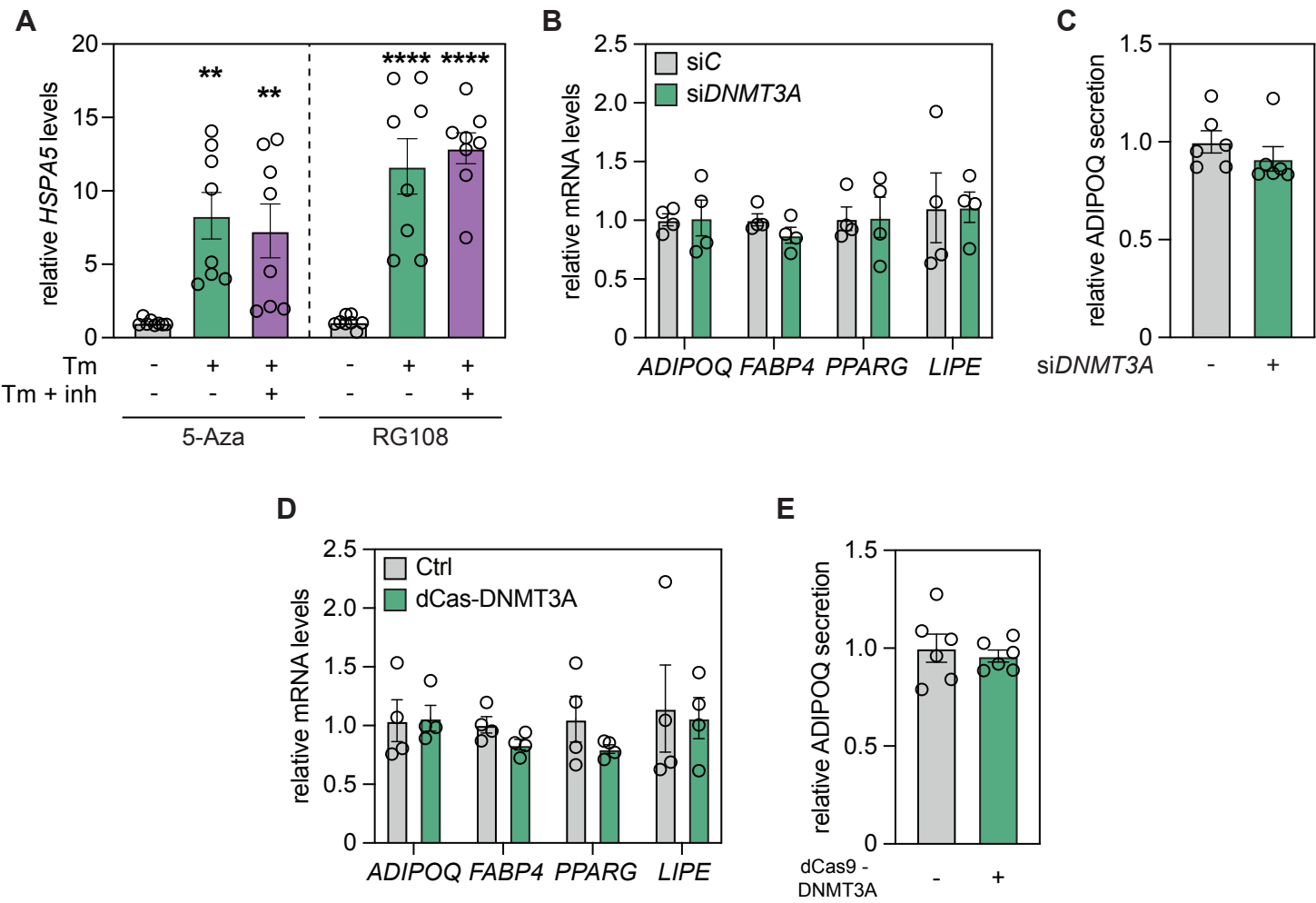

Supplement: Multimedia component 7 [file mmc7.pdf]

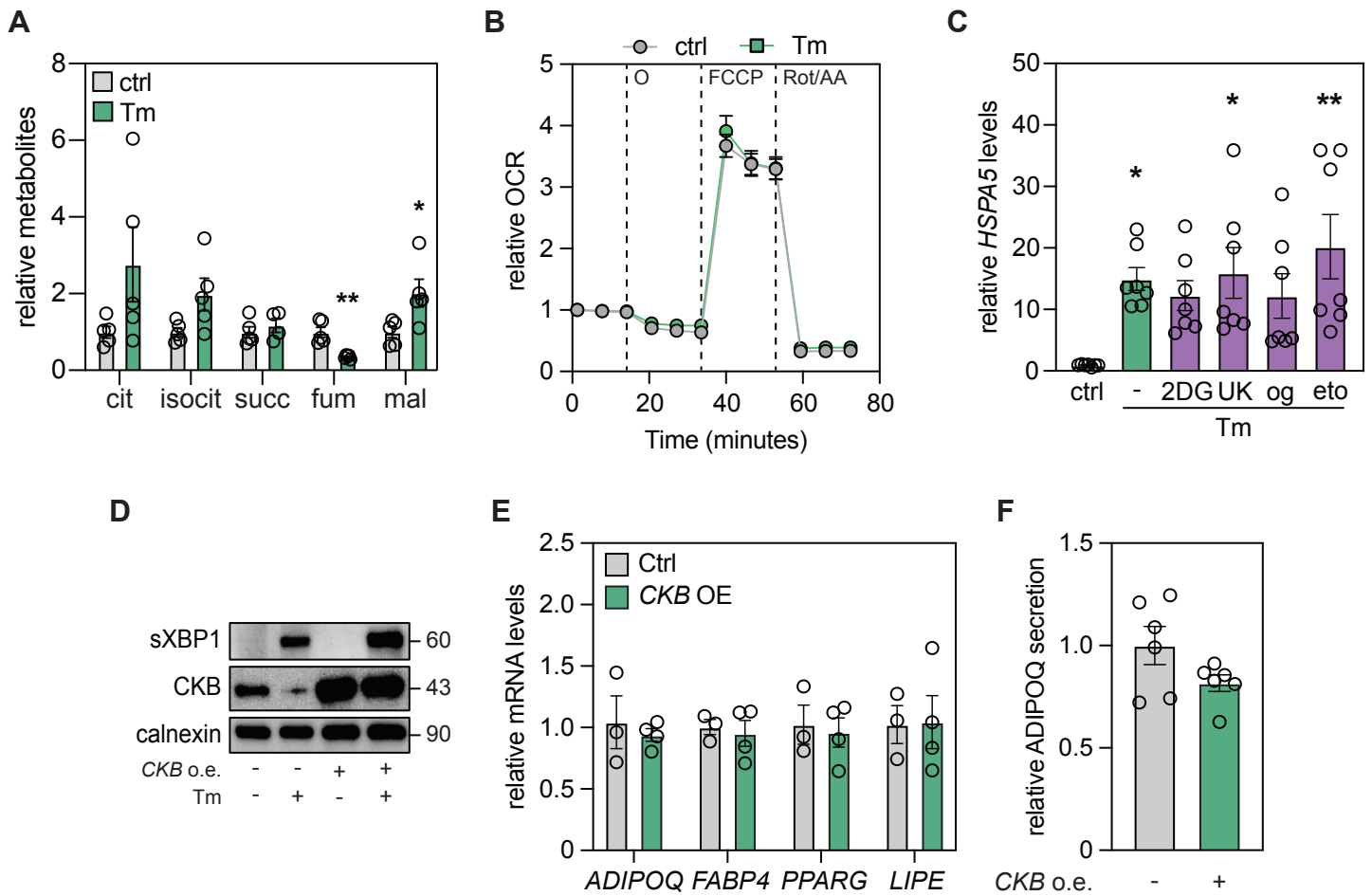

Supplement: Multimedia component 8 [file mmc8.pdf]
