# Supplementary material for: Epigenetic suppression of creatine kinase B in adipocytes links endoplasmic reticulum stress to obesity-associated inflammation
Source: Mol Metab. 2024 Dec 13;92:102082. doi: 10.1016/j.molmet.2024.102082 (PMC11731883; doi:10.1016/j.molmet.2024.102082)
Supplement: Multimedia component 9 [file mmc9.docx]

**Supplementary figure legend**

**Figure S1.** Gene expression of *CKB* and *HSPA5* in cells incubated with or without palmitate (palm.). Values are mean ± SEM. *P* value calculated by a student’s two-tailed t-test assuming unequal variance.

Statistical significance is presented as following: *P* < 0.05 *, *P* < 0.01 **, *P* < 0.001 ***, *P* < 0.0001 ****.

**Figure S2. A.** *HSPA5* gene expression in cells with or without Tm, and 4μ8C, GSK or CA7. Values are mean ± SEM. Statistical significance was calculated using one-way ANOVA comparing all the conditions to control. **B.** Effects on *CKB* and *HSPA5* expression following treatment with Tm and a combination of 4μ8C, GSK and CA7, as indicated. Representative example from one experiment run in triplicates. **C.** Gene expression of *Ckb* and *Hspa5* in 3T3-L1 cells incubated with or without Tm and 4μ8C. Values are mean ± SEM. Statistical significance was calculate using One-Way ANOVA comparing all the conditions to control.  **D.** Gene expression of *HSPA5* from si*C* or si*XBP1* transfected cells, incubated with or without Tm. Values are mean ± SEM. Statistical significance was calculate using One-Way ANOVA comparing all the conditions to control. **E-F.** Gene expression of adipocyte markers (**E**) and adiponectin secretion (**F**) from si*C* or si*XBP1* transfected cells. **G.** Gene expression of total *XBP1* in cells transfected with mRNA encoding *XBP1s* after 24 hours. Splicing of *XBP1*, is displayed underneath the plot. Values are mean ± SEM. *P* value calculated by a student’s two-tailed t-test assuming unequal variance. **H-I.** Gene expression of adipocyte markers (**H**) and adiponectin secretion (**I**) from control cells and adipocytes transfected with XBP1 mRNA.

Statistical significance is presented as following: *P* < 0.05 *, *P* < 0.01 **, *P* < 0.001 ***, *P* < 0.0001 ****.

**Figure S3.** **A.** Gene expression of *HSPA5* in cells incubated with or without Tm, together with 5-Azacytidine or RG108. Values are mean ± SEM. Statistical significance was calculate using One-Way ANOVA comparing all the conditions to control. **B-C.** Gene expression of adipocyte markers (**B**) and adiponectin secretion (**C**) from si*C* or si*DNMT3A* transfected adipocytes. **D-E.** Gene expression of adipocyte markers (**D**) and adiponectin secretion (**E**) from control cells and adipocytes overexpressing catalytically inactive Cas9 fused to DNMT3A.

Statistical significance is presented as following: *P* < 0.05 *, *P* < 0.01 **, *P* < 0.001 ***, *P* < 0.0001 ****.

**Figure S4. A.** TCA related metabolites of cells incubated with or without Tm. Values are mean ± SEM. *P* value calculated by a student’s two-tailed t-test assuming unequal variance. **B.** Representative relative oxygen consumption rate of cells incubated with or without Tm. Values are mean ± SEM. **C.** Gene expression of *HSPA5* in cells incubated with/without Tm in concomitance with selective inhibitors of intracellular metabolic pathway (2-Deoxy Glucose, UK5099, Oligomycin, Etomoxir). Values are mean ± SEM. Statistical significance was calculated using One-Way ANOVA comparing all the conditions to control. **D.** Western blots for XBP1s, CKB, and Calnexin (ER marker), from cells transfected with *CKB* mRNA with or without Tm. Representative example from one out of three independent experiments. **E-F.** Gene expression of adipocyte markers (**E**) and adiponectin secretion (**F**) from control cells and adipocytes overexpressing *CKB*.

Statistical significance is presented as following: *P* < 0.05 *, *P* < 0.01 **, *P* < 0.001 ***, *P* < 0.0001 ****.
